# Heart failure labelled patients with missing ejection fraction in primary care: prognosis and determinants

**DOI:** 10.1186/s12875-017-0612-6

**Published:** 2017-03-17

**Authors:** Miguel-Angel Muñoz, Xavier Mundet-Tuduri, Jordi Real, José-Luis Del Val, Mar Domingo, Ernest Vinyoles, Ester Calero, Caterina Checa, Nuria Soldevila-Bacardit, José-María Verdú-Rotellar

**Affiliations:** 1 Institut Català de la Salut, Barcelona, Spain; 2 Primary Healthcare University Research Institute IDIAP-Jordi Gol, Barcelona, Spain; 3 Universitat Autònoma de Barcelona, Bellaterra, Spain; 4 EAP Dreta de l’Eixample, Barcelona, Spain; 5 Universitat de Barcelona, Barcelona, Spain; 6 Epidemiologia i Salut Pública, Universitat Internacional de Catalunya, Sant Cugat, Spain

**Keywords:** Primary care, Heart failure, Echocardiography, Diagnosis

## Abstract

**Background:**

It is common to find a high variability in the accuracy of heart failure (HF) diagnosis in electronic primary care medical records (EMR). Our aims were to ascertain (i) whether the prognosis of HF labelled patients whose ejection fraction (EF) was missing in their EMR differed from those that had it registered, and (ii) the causes contributing to the differences in the availability of EF in EMR.

**Methods:**

Retrospective cohort analyses based on clinical records of HF and attended at 52 primary healthcare centres of Barcelona (Spain). Information of 8376 HF patients aged > 40 years followed during five years was analyzed.

**Results:**

EF was available only in 8.5% of primary care medical records. Cumulate incidence for mortality and hospitalization from 1st January 2009 to 31th December 2012 was 37.6%. The highest rate was found in patients with missing EF (HR 1.84, 95% CI 1.68 -1.95) compared to those with preserved EF. Patients hospitalized the previous year and those requiring home healthcare (HR 1.81, 95% Confidence Interval 1.68-1.95 and HR 1.58, 95% CI 1.46-1.71, respectively) presented a higher risk of having an adverse outcome. Older patients, those more socio-economically disadvantaged, obese, requiring home healthcare, and taking loop diuretics were less likely to have an EF registered.

**Conclusions:**

EF is poorly recorded in primary care. HF patients with EF missing at medical records had the worst prognosis. They tended to be older, socio-economically disadvantaged, and more fragile.

## Background

It is well established that heart failure (HF) symptoms, especially during the early stages, are not specific. This is particularly evident in obese and elderly populations, and in patients suffering from chronic pulmonary diseases [1–3]. Up to 60% of HF patients are not properly diagnosed, and as many as 38% who are HF registered have not had an echocardiogram registered in their medical records [4, 5]. We are unaware of the causes related to this lack of information. Two studies have shown that some General Practitioners did not take in account EF to diagnose HF [6, 7]. It is, therefore, difficult to properly estimate the prognosis and evaluate the efficacy of evidence-based treatment in a large number of HF patients, especially since much of the data comes from clinical trials in which the population has been strictly selected.

Prior hospitalization as a consequence of HF has been considered a valid criterion to confirm diagnosis. Nevertheless, it is possible that gaps exist in sharing information between the hospital and primary care setting which may lead to under registration of HF cases in the primary care Electronic Medical Records (EMR).

Most HF patients, especially the oldest ones and those with multimorbidity, are mainly managed in the primary care setting [8] and, especially those in terminal phases of their disease, are not eligible to be referred to a specialist for an echocardiography.

Regarding the validity of the diagnosis, Schultz et al., argued that if a physician is treating a patient as having HF it is reasonable to consider that this patient is properly labelled HF [9].

Considering the ejection fraction (EF) as a measure to estimate prognosis has proven controversial [10, 11] a recent meta-analysis found lower mortality in HF patients with preserved ejection fraction (HF-PEF) than in those with reduced ejection fraction (HF-REF) [12]. In addition, it has been reported that in patients with unknown EF (i.e. unrecorded) mortality is similar to those with HF-REF and higher in those with HF-PEF [13].

Our study is aimed at analysing the different prognoses of patients registered as HF in primary healthcare records, depending on whether they have HF- REF, HF-PEF, or Possible HF (HF labelled patients with missing ejection fraction), and, if possible, to ascertain the causes contributing to the differences in EF availability in the EMR.

## Methods

The present study is a retrospective cohort analysis with four year follow-up. It is based on the clinical information included in the EMR of all HF patients labelled at the 52 primary healthcare centres of the Institut Català de la Salut in Barcelona (Spain).

Clinical information is centralized in the SIDIAP database (Information System for the Development of Research in Primary Care). This database has been shown to be a valid source for cardiovascular disease research [13] and is linked to the Catalan hospital discharge database CMBD-AH (Conjunto Mínimo Básico de Datos de Altas Hospitalarias) [14].

All adult patients >40 years living in Barcelona (Spain), who were labelled HF diagnosis (International Classification Diseases: I.50) registered in their primary EMR on December 31st, 2012, were included.

The registration date of the HF labelling in the EMR was considered as the date of study inclusion. The duration of the study was from the 1st January, 2009 to 31st December, 2012.

Prognosis was determined by hospital admission as a consequence of HF and the global mortality that occurred during the study period.

Event-free time was defined as the period between diagnosis registration and the first hospital admission as a consequence of HF, global mortality, or the last contact with the primary care services.

**Fig. 1:**
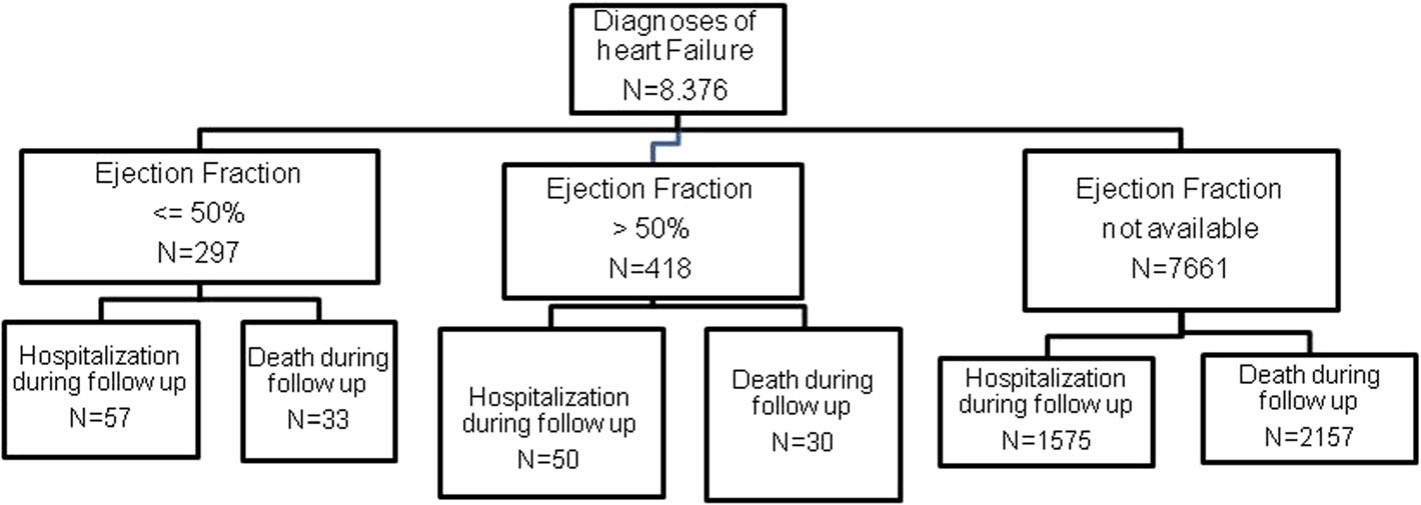
Classification of heart failure patients according to ejection fraction in primary care electronic medical records

*Ejection Fraction*: in order to be able to compare our results with previously published studies, HF patients were classified into three categories according to the EF closest to the date of the inclusion: HF-REF (EF < 50%), HF-PEF (EF > = 50%), and Possible HF (when no information, either quantitative or qualitative, about EF was found in the medical records) (Fig. 1).

The following potentially confounding variables at baseline for EF effect were considered: age, gender, hospitalization for HF the year prior to inclusion in the study, smoking habit, cardiovascular risk factors (hypertension, diabetes, hypercholesterolemia, obesity), cardiovascular comorbidity (coronary heart disease, atrial fibrillation, stroke, peripheral arterial disease), other comorbidities (chronic obstructive pulmonary disease, chronic nephropathy), cardiovascular drug use (antiagregants, lipid lowering drugs, beta-blockers, angiotensin converting-enzyme inhibitors or angiotensin receptor blockers, and loop diuretics). We also recorded whether the patients required domiciliary healthcare. Socio-economic levels were measured by the MEDEA index which categorizes populations in quintiles, the first one representing the least disadvantaged. This index is based on several items (unemployment, percentage of manual and temporary workers and persons with insufficient education overall and in young people) [15].

The whole population registered as HF in the primary care EMR in Barcelona (Spain) was included in the analysis, resulting in a sample of 8376 HF patients. These sample reflects the whole population labelled as HF.

In 52 participating primary healthcare centres were registered more than one million subjects. Such a sample size reaches 94% statistical power (estimated power for Cox regression with Wald test; alpha = 0.05 two sides) with a minimum of 30% events observed. It thus ensures enough statistical power to answer the main questions of the study.

Data are expressed as frequencies and percentages for categorical variables, and means (standard deviation) for continuous ones. Baseline homogeneity of variables according to the EF was analysed. The one-way ANOVA, and Chi-square tests were used to evaluate differences amongst groups with different or missing EF.

Cumulate incidence of mortality, hospital admission or a combined variable of both events during the follow-up period was estimated for each group (HF-REF, HF-PEF, and Possible HF).

To evaluate the differences among groups according to the time from the inclusion date, Cox regression models crude and adjusted were made. The Hazard Ratio (HR) of each group with respect to the reference was calculated and 95% confidence intervals (95%CI) were estimated to compare the groups. The models were compiled using the Enter Method, including clinically relevant co-variables and those statistically associated with the previous EF (< or equal to 50%, > 50%, or not available). We evaluated goodness-of-fit and the Cox model’s proportional risk assumption, as well as the interactions at different levels of exposure to each drug, using the Schoenfeld residual analysis. Furthermore, to evaluate the factors related to the probability of having an EF, multivariable logistic regression was performed. For all analyses, a two-tailed p < 0.05 was considered statistically significant. IBM-SPSS PC v21 package was used to perform statistical analysis.

## Results

From 1st January, 2009 to 31st December 2012, a total of 8376 patients labelled with HF met study criteria,. Median follow-up to event or end of the study was 16.3 months. During the study period, 1608 (19.2%) patients died and 2264 (27.0%) were hospitalized.

Women represented 55.9% of patients and mean age of the population was 78.0 (SD 10.2) years.

Among the sample, 3013 patients (36%) had been admitted to hospital during the year prior to inclusion in the study as a consequence of HF. Ejection fraction was available only in 8.5% of the EMR.

The flow chart represents the distribution of outcomes according to EF (Fig. 1).

By comparing the three categories according to EF, patients in the group with HF-REF were predominantly men (67.0%), had been hospitalized as a consequence of HF the year prior to inclusion in the study (43.8%), and suffered more frequently from coronary heart disease (42.7%). These patients were more often treated with ACE inhibitors and beta-blockers (85.5 and 69.9%, respectively).

Patients with Possible HF were older, required home healthcare, obese, and more frequently treated with loop diuretics. With the exception of coronary disease, no differences were observed in the proportion of patients with atrial fibrillation, stroke history, peripheral artery disease, chronic pulmonary disease, and chronic renal failure according to the EF among the three categories.

The use of ACE inhibitors and beta- blockers was very similar in the group of HF-PEF and Possible HF. The highest proportion of hospitalized or died patients was in the group of Possible HF (39.1%) (Table 1).

**Table 1:** Characteristics, clinical profile, and treatment according to the ejection fraction of heart failure patients

	EF >50 *n*=418		EF <=50 *n*=297		No EF *n*=7661		EF>50 versus No EF	EF < =50 versus No EF
	n	%	N	%	n	%	*p* value	*p* value
Gender (women)	253	60,5	98	33,0	4331	56,5	0,109	<0.001
Age (Mean,SD)	77.1 (9.5)		74.2 (11.5)		78.3 (10.2)		0.053	<0.001
Socioeconomic deprivation index								
1 (less disadvantaged)	98	23.4	60	20.2	1486	19.4		
2	126	30.1	97	32.6	1377	17.9		
3	102	24.4	53	17.8	1533	20.0		
4	60	14.3	48	16.1	1544	20.1		
5 (more disadvantaged)	29	6.9	36	12.1	1586	20.7	<0.001	<0.001
Previous hospitalization for heart failure	91	21.8	130	43.8	2792	36.4	<0.001	0,010
Patients requiring home care	64	15.3	54	18.1	2124	27.7	<0.001	<0.001
Cardiovascular risk factors								
Hypertension	328	78.4	214	72.0	5823	76.0	0,250	0,118
Diabetes	129	30.8	108	36.3	2528	33.0	0,365	0,227
Hypercholesterolemia	145	34.6	97	32.6	2241	29.2	0,018	0,206
Obesity	71	16.9	42	14.1	1742	22.7	0,006	<0.001
Smoking habit	26	6.2	33	11.1	619	8.0	0,380	<0.001
Cardiovascular disease								
Coronary heart disease	94	22.4	127	42.7	2009	26.2	0,090	0,000
Atrial fibrillation	150	35.8	98	33.0	2794	36.4	0,809	0,222
Stroke	37	8.8	30	10.1	770	10.0	0,426	0,978
Peripheral artery disease	26	6.2	24	8.0	463	6.0	0,883	0,151
COPD	65	15.5	49	16.50	1240	16.19	0,731	0,886
Chronic renal failure	83	19.8	53	17.85	1397	18.24	0,404	0,864
Drug therapy								
ACE inhibitor or ARB	305	73.0	254	85.5	5736	74.9	0,382	<0.001
Beta-blocker	212	50.7	205	69.0	3729	48.7	0,416	<0.001
Loop diuretics	246	58.8	212	71.38	5889	76.8	<0.001	0,028
Outcomes								
Hospitalization	50	11,96	57	19,1	2157	28,1	0,000	0,001
Mortality	30	7,18	33	11,1	1545	20,1	0,000	0,000

*EF* Ejection Fraction, *SD* Standard deviation, *COPD* Chronic obstructive pulmonary disease, *ACE* angiotensin converting enzyme, *ARB* Angiotensin Receptor Blocker

Table 2 shows the crude and adjusted hazard ratios for hospitalization or death. Cumulate incidence was 37.6%, and the highest rate was found in patients with Possible HF.

**Table 2:** Cumulate incidence and hazard ratio, crude and adjusted, for hospitalization or death, with respect to socio demographic characteristics, clinical profile, and treatment received for heart failure

	Cumulate Incidence		Crude Hazard Ratio		Adjusted Hazard Ratio	
	Rate (%)	95% Confidence interval	HR	95% Confidence interval	HR	95% Confidence interval
Goblal incidence	37.6	36.5-38.7				
Ejection Fraction						
Ejection Fraction>50	17.0	13.3-20.7	1		1	
Ejection Fraction <=50	27.3	22.1-32.4	1.81	1.31-2.49	1.36	0.99-1.88
Missing Ejection Fraction	39.1	38.0-40.2	2.33	1.84-2.95	1.84	1.45-2.33
Previous hospitalization for heart failure	51.3	49.5-53.1	2.18	2.03-2.34	1.81	1.68-1.95
Gender (Male)	39.8	38.2-41.4	1.15	1.07-1.23	1.24	1.14-1.34
Age (quintiles)						
(<=71 years; reference	29.0	26.8-31.1	1		1	
72 - 78	33.3	31.2-35.5	1.14	1.02-1.28	1.03	0.91-1.16
79 - 82	38.1	35.6-40.5	1.43	1.27-1.61	1.21	1.07-1.37
3 - 86	41.1	38.5-43.6	1.62	1.44-1.82	1.30	1.14-1.48
>=87	48.8	46.2-51.3	2.17	1.94-2.43	1.60	1.40-1.83
Socio economic deprivation index						
1 (less disadvantaged)	33.5	31.1-35.8	1		1	
2	36.7	34.3-39.1	1.08	0.96-1.21	1.10	0.98-1.24
3	37.3	34.9-39.6	1.06	0.95-1.19	1.01	0.90-1.13
4	38.6	36.2-41.0	1.11	0.99-1.25	1.03	0.92-1.16
5 (more disadvantaged)	42.8	40.4-45.3	1.28	1.14-1.43	1.13	1.01-1.27
Patients requiring home care	57.0	55.0-59.1	2.13	1.98-2.29	1.58	1.46-1.71
Cardiovascular risk factors						
Hypertension	38.3	37.1-39.5	1.13	1.04-1.22	1.08	0.99-1.19
Diabetes	41.6	39.7-43.5	1.25	1.16-1.34	1.17	1.08-1.26
Hypercholesterolemia	34.5	32.6-36.4	0.90	0.83-0.97	0.94	0.86-1.02
Obesity	37.7	35.5-40.0	0.99	0.91-1.08	1.00	0.92-1.10
Smoking habit	39.2	35.5-43.0	0.98	0.87-1.11	1.04	0.91-1.19
Cardiovascular diseases						
Coronary heart disease	41.5	39.4-43.6	1.21	1.12-1.31	1.11	1.02-1.21
Atrial fibrillation	42.4	40.6-44.2	1.31	1.22-1.41	1.05	0.96-1.15
Stroke	44.7	41.2-48.1	1.41	1.26-1.57	1.15	1.03-1.29
Peripheral artery disease	45.4	41.0-49.8	1.45	1.27-1.66	1.11	0.97-1.28
Other comorbidities						
Chronic obstructive pulmonary disease	45.1	42.3-47.8	1.39	1.28-1.52	1.25	1.14-1.37
Chronic renal failure	44.8	42.3-47.4	1.47	1.35-1.60	1.18	1.08-1.29
Drug therapy						
ACE inhibitor or ARB*	37.3	36.1-38.5	0.91	0.84-0.99	0.85	0.78-0.93
Beta-blocker	37.9	35.3-40.5	1.00	0.91-1.09	1.03	0.94-1.14
Loop diuretics	42.5	41.3-43.8	2.27	2.05-2.51	1.64	1.48-1.82
Statins	35.1	33.6-36.6	0.87	0.81-0.94	0.85	0.79-0.93

^a^ *ACE* angiotensin converting enzyme, *ARB* Angiotensin Receptor Blocker

The oldest patients presented 60% more risk (HR: 1.6; CI95%:1.40-1.83) of having an adverse event than the younger ones (≤71 years). Being hospitalized as a consequence of a decompensation the year prior to inclusion almost doubled the risk for re-hospitalization or death (HR: 1.81; CI95%:1.68-1.95). In addition, patients requiring home healthcare had 60% (HR:1.58; CI95:1.46-1.71) more risk than the others of having an adverse event. This risk was also higher among patients living in socio-economically disadvantaged neighborhoods (HR:1.13; CI95%:1.01-1.27).

Hypertension, diabetes, pulmonary and renal diseases, and cardiovascular comorbidity were also associated with a higher risk of having an adverse outcome. Medication for hypercholesterolemia and hypertension, however, worked as protective factors. In contrast, patients taking loop diuretics had a higher rate of adverse outcomes.

Patients hospitalized the year before and without an EF registered in the EMR presented an HR of 4.99, and a 95% Confidence Interval 3.67 to 6.78, for being hospitalized or dying during follow-up.

The adjusted analysis to identify the causes related to the higher probability of missing an EF in the medical records showed that among patients who were elderly, more socio-economically disadvantaged, obese, requiring home healthcare, and taking loop diuretics it was less common to have one registered (Table 3).

**Table 3:** Factors related to the probability of having an ejection fraction registered in electronic medical records

	OR^a^	95% Confidence interval
Age (quintiles)		
<=71 años (reference)	1	
72 - 78	0.86	0.68-1.08
79 - 82	0.71	0.55-0.92
83 - 86	0.81	0.63-1.05
87+	0.50	0.37-0.68
Socio economic deprivation index		
1 (less disadvantaged)	1	
2	1.55	1.24-1.94
3	0.99	0.78-1.25
4	0.68	0.52-0.88
5 (more disadvantaged)	0.41	0.30-0.56
Patients requiring home care	0.71	0.56-0.88
Obesity	0.68	0.55-0.85
Drug therapy for heart failure		
ACE inhibitor or ARB^b^	1.25	1.02-1.53
Beta-blockers	1.23	1.01-1.51
Loop diuretics	0.59	0.49-0.71

^a^Odds ratio adjusted by gender, hypertension, diabetes, hypercholesterolemia, smoking habit, coronary heart disease, atrial fibrillation, stroke, peripheral artery disease, and previous hospitalization for heart failure

^b^ *ACE* angiotensin converting enzyme, *ARB* Angiotensin Receptor Blocker

## Discussion

Our study found that patients labelled with HF who did not have an EF registered in their primary care EMR presented the highest rates of death and hospital re-admissions with respect to those who did. Patients hospitalized as a consequence of HF decompensation the year prior to study inclusion presented a higher probability of having an adverse outcome during follow- up.

The use of administrative databases could be a limitation to properly answer some research questions. The difficulty of having an accurate HF diagnosis registered is well known. Although it has been reported that most HF diagnoses registered in EMR correspond to authentic cases, about one-quarter are not recorded [16].

On the other hand, the use of a large primary care database allows us to have information about the whole population and confers a high external validity. This validity has been previously analyzed and found good for the study of cardiovascular diseases [13]. Although MEDEA deprivation index is not an individual measure but an ecological one, it is useful as a proxy to determine socioeconomic position of the population living in a geographical area.

The MAGGIC study had already reported higher mortality in Possible HF patients, but their proportion of missing EF was lower in our study and some questions were not completely answered, such as socioeconomic position and setting of care (ambulatory or home healthcare, and). In this way we found that patients requiring home care and those in more disadvantaged economic position had a higher probability of not having a EF registered at their EMR.

On the other hand, Possible HF patients had up to 50% more probability of having adverse outcomes than those with HF-REF, and the risk doubled with respect to HF-PEF patients [13].

We have identified several factors which could help explain these findings. Firstly, patients who lacked information about EF were older, as has been reported by other authors in patients attended for acute heart failure at hospital emergency rooms [17]. In our population, the probability for the oldest patients of having an EF recorded in their EMR was less than 50% with respect to the others.

Socio-economic inequality regarding access to specialized care in newly diagnosed HF patients, and a lower probability of undergoing invasive cardiac procedures for the less disadvantaged populations, have been previously described [18, 19]. Nevertheless, most evidence comes from countries with differing healthcare systems where accessibility could vary. In contrast, the Spanish National Health System provides healthcare universal and free. Previous studies performed by our group did not find any inequality regarding therapeutic management in populations suffering from coronary heart disease [20] or at high cardiovascular risk [21, 22].

We, cannot, therefore, satisfactorily account for the fact that the more socio- economically deprived patients showed a lower probability of having an echocardiography performed.

Due to their deteriorated health, patients needing home care are not usually candidates to be referred to undergo tests and explorations, including EF measures. As a consequence, the probability of having an echocardiography is lower than in those with a better life expectancy. Home healthcare is generally oriented towards achieving a better quality of life rather than actually lengthening it. In addition, patients needing this service usually have a very limited quality of life and the hypothetical availability of their EF figures would probably not result in treatment changes. A recent experience in united Kingdom showed that a program of basic cardiac scans (‘Quick scans’) performed in highest risk population could reduce the demand of echocardiography and optimize the detection of structural disease [23].

In agreement with other authors, we found that previous history of HF hospitalization, especially in the previous year, is a powerful predictor for having recurrent events [24–26].

In addition, and again concurring with other publications, we observed that the use of loop diuretics was associated with a higher risk of mortality and hospitalization in HF patients. It has been argued that this effect occurs especially when doses are high and glomerular filtration declines [27].

## Conclusions

EF is poorly recorded in primary care. HF patients with EF missing at medical records had the worst prognosis with regard to hospitalisation and survival. They tended to be older, socio-economically disadvantaged, and more fragile.

## Abbreviations

CMBD-AH: Conjunto Mínimo Básico de Datos de Altas Hospitalarias
EF: Ejection fraction
EMR: Electronic Medical Record
HF: Heart failure
HF-PEF: Heart failure patients with preserved ejection fraction
HF-REF: Heart failure patients with reduced ejection fraction
HR: Hazard ratio
SIDIAP: Information System for the Development of Research in Primary Care
